# Cardiovascular manifestations of renovascular hypertension in diabetic mice

**DOI:** 10.7717/peerj.1736

**Published:** 2016-02-22

**Authors:** Sonu Kashyap, Sean Engel, Mazen Osman, Yousif Al-Saiegh, Asarn Wongjarupong, Joseph P. Grande

**Affiliations:** 1 Department of Laboratory Medicine and Pathology, Mayo Clinic, Rochester, MN, USA; 2 Wartburg College, Waverly, IA, United States; 3 Hannover Medical School (MHH), Hannover, Germany; 4 Department of Medicine, Chulalongkorn University, Bangkok, Thailand; 5 Division of Nephrology and Hypertension, Mayo Clinic, Rochester, MN, USA

**Keywords:** Renal artery stenosis, Cardiovascular disease, Diabetes, Hypertension

## Abstract

**Purpose.** Type 2 diabetes is the leading cause of end stage renal disease in the United States. Atherosclerotic renal artery stenosis is commonly observed in diabetic patients and impacts the rate of renal and cardiovascular disease progression. We sought to test the hypothesis that renovascular hypertension, induced by unilateral renal artery stenosis, exacerbates cardiac remodeling in leptin-deficient (db/db) mice, which serves as a model of human type II diabetes.

**Methods.** We employed a murine model of renovascular hypertension through placement of a polytetrafluoroethylene cuff on the right renal artery in db/db mice. We studied 109 wild-type (non-diabetic, WT) and 95 db/db mice subjected to renal artery stenosis (RAS) or sham surgery studied at 1, 2, 4, and 6+ weeks following surgery. Cardiac remodeling was assessed by quantitative analysis of the percent of myocardial surface area occupied by interstitial fibrosis tissue, as delineated by trichrome stained slides. Aortic pathology was assessed by histologic sampling of grossly apparent structural abnormalities or by section of ascending aorta of vessels without apparent abnormalities.

**Results.** We noted an increased mortality in db/db mice subjected to RAS. The mortality rate of db/db RAS mice was about 23.5%, whereas the mortality rate of WT RAS mice was only 1.5%. Over 60% of mortality in the db/db mice occurred in the first two weeks following RAS surgery. Necropsy showed massive intrathoracic hemorrhage associated with aortic dissection, predominantly in the ascending aorta and proximal descending aorta. Aortas from db/db RAS mice showed more smooth muscle dropout, loss of alpha smooth muscle actin expression, medial disruption, and hemorrhage than aortas from WT mice with RAS. Cardiac tissue from db/db RAS mice had more fibrosis than did cardiac tissue from WT RAS mice.

**Conclusions.** db/db mice subjected to RAS are prone to develop fatal aortic dissection, which is not observed in WT mice with RAS. The db/db RAS model provides the basis for future studies directed towards defining basic mechanisms underlying the interaction of hypertension and diabetes on the development of aortic lesions.

## Introduction

Diabetes, hypertension, and hyperlipidemia are major risk factors for the development of cardiovascular disease, the leading cause of death in the United States (Lee et al., 2014). Diabetes is the most common cause of chronic renal disease, and is responsible for up to 50% of end stage renal disease cases in developed countries (Tuttle et al., 2014). In addition to increased risk for myocardial infarction and stroke, patients with diabetes are prone to develop a diabetic cardiomyopathy, characterized by extensive fibrotic changes and cardiomyocyte hypertrophy, leading to increased myocardial stiffness and diastolic dysfunction (Asbun & Villarreal, 2006; Rydén et al., 2013).

Hypertension is a major risk factor for both renal disease progression and cardiovascular morbidity and mortality in patients with type 2 diabetes (Ghaderian et al., 2015). Atherosclerotic renal artery stenosis is one of the most common causes of secondary hypertension (Safian & Textor, 2001). The prevalence of renal artery stenosis approaches 7% in individuals greater than 65 years of age and is up to 45% in patients with coronary artery or aortoiliac disease (Hansen et al., 2002; Weber-Mzell et al., 2002; Iglesias et al., 2000). The prevalence of renal artery stenosis varies from 17–44% in patients with hypertension and diabetes (Valabhji et al., 2000).

It is well recognized that renal artery stenosis promotes cardiac remodeling, characterized by replacement of myocardial tissue with extracellular matrix (Khan et al., 2014; Al-Suraih & Grande, 2014). However, mechanisms underlying the additive or synergistic effects of renovascular hypertension and cardiac remodeling have not been adequately addressed in previous studies. In order to address this issue, we have established a murine model of diabetic renovascular disease through placement of a cuff on the right renal artery of leptin deficient mice (db/db mice), which develop obesity and type 2 diabetes (Hartono et al., 2014).

Throughout the course of our studies, we found an increased incidence of sudden death in db/db mice subjected to renal artery stenosis (RAS), which was not observed in wild-type mice subjected to RAS. Necropsy of mice available for analysis revealed massive hemothorax and/or hemoperitoneum, which was associated with aortic dissection.

The objective of this study was to characterize the aortic and cardiac phenotype of db/db mice subjected to RAS. We found that cardiac tissue from db/db RAS mice had more fibrosis than did cardiac tissue from WT RAS mice studied at greater than 2 weeks following surgery. Aortas from db/db RAS mice had more smooth muscle dropout, medial disruption, and hemorrhage than did aortas from WT mice with RAS at both early (less than 2 weeks) and late time points (greater than 2 weeks). The db/db RAS model provides the basis for future studies directed towards defining basic mechanisms underlying the interaction of hypertension and diabetes on the development of aortic lesions.

## Methods

### Animal model

For survival analysis, a total of C57BLKS (WT) (*N* = 109) and C57BLKS/JLepr (db/db) (*N* = 95) male mice, (Jackson Laboratory, Bar Harbor, ME, USA) were studied. Both WT and db/db mice at 6–7 weeks age underwent RAS or sham surgery through placement of a polytetrafluoroethylene cuff (0.2 mm internal diameter) on the right renal artery, as previously described (*N* = 68 for WT and *N* = 64 db/db) (Warner et al., 2012; Wang et al., 2013). Sham surgery was performed through manipulation of the right renal artery without placement of the cuff (*N* = 41 for WT and *N* = 31 db/db). Mice were sacrificed at 1 week (*N* = 33 WT RAS, *N* = 22 WT sham, *N* = 18 db/db RAS, *N* = 5 db/db sham), 2 weeks (*N* = 8 WT RAS, *N* = 5 WT sham, *N* = 17 db/db RAS, *N* = 8 db/db sham), 4 weeks (*N* = 10 WT RAS, *N* = 5 WT sham, *N* = 8 db/db RAS, *N* = 5 db/db sham), 6 weeks (*N* = 10 WT RAS, *N* = 5 WT sham, *N* = 13 db/db RAS, *N* = 8 db/db sham), and 17 weeks (*N* = 7 WT RAS, *N* = 4 WT sham, *N* = 8 db/db RAS, *N* = 5 db/db sham).

For analysis of aortas and heart, a total of 38 WT RAS (*N* = 18 harvested at week 1, *N* = 3 week 4, *N* = 10 week 6, and *N* = 7 week 17), 19 WT sham (*N* = 5 week 1, *N* = 5 week 2, *N* = 5 week 6, and *N* = 4 week 17), 48 db/db RAS (*N* = 11 week 1, *N* = 10 week 2, *N* = 7 week 4, *N* = 12 week 6, *N* = 8 week 17), and 22 db/db sham (*N* = 5 week 1, *N* = 3 week 2, *N* = 9 week 6, and *N* = 5 week 17) were studied. The animals studied were divided into early time point (pre 2 weeks following surgery) and late time point (post 2 weeks following surgery) groups.

All animal protocols were approved by the Mayo Clinic Institutional Animal Care and Use Committee for appropriate experiments (IACUC Protocol Number A62613).

### Histological and Immunohistochemical analysis

Aortas were carefully examined for grossly apparent abnormalities, including dilation, medial disruption, or hemorrhage. Five aortas, obtained from db/db RAS mice that died suddenly, showed evidence of aortic dissections involving the ascending or proximal descending aorta. If there were no grossly apparent abnormalities, a section of ascending aorta, taken from the same site among samples, was submitted for histologic processing. Aorta and heart tissues were fixed with 10% neutral buffered formalin and then processed for histology or immunohistochemistry using standard techniques. Histological sections of heart and aorta (5 µm thick) were stained with hematoxylin-eosin (H & E). H & E was used for scoring the aorta pathology and aortic diameter. The aortic score numbers were represented as, 0 = normal aorta; 1 = isolated smooth muscle dropout; 2 = multifocal smooth muscle dropout; 3 = hemorrhage, necrosis, dissection, or mural thrombosis. Slides were read in a blinded fashion. Heart sections were also stained with Masson’s trichrome stain and used for the quantification of fibrosis. Width of aorta and percentage of fibrosis in heart trichrome sections were quantified at 200× magnification using an Olympus BX50 microscope (Olympus America, Melville, NY, USA), a Micropublisher 3.3 RTV camera (Q-Imaging, Surrey, BC, CAN), and the NIS Elements Imaging Software (Nikon Instruments, Inc., Melville, NY, USA).

Sections of aortas were stained for alpha smooth muscle actin (1:500, Abcam Inc., Cambridge, MA, USA). Loss of alpha smooth muscle actin in the media of aortas was semiquantitatively assessed as 0 = none, 1 = isolated, 2 = multifocal, and 3 = generalized. Assessment was conducted in a blinded fashion.

### Statistical analysis

Data are presented as mean ± SEM. Comparisons between two groups were done using student *t*-test for parametric data and Mann–Whitney test for nonparametric data. For comparison across multiple groups, one-way ANOVA followed by a Turkey adjustment was used for post-hoc comparison of the measurements. *P* values <0.05 were considered significant. Correlation analysis and all statistical comparisons were performed using Graphpad Prism 6 (GraphPad Software, La Jolla, CA, USA).

## Results

A summary of heart weight, body weight and heart-to-body weight ratio at time of analysis is provided in Table 1. In accordance with our previous studies, both db/db RAS and WT RAS mice became hypertensive within 1 week and remained hypertensive thereafter, with no significant differences between db/db RAS and WT RAS at any time point (Hartono et al., 2014). As expected, the weight of both db/db sham and db/db RAS mice was significantly greater than that of WT sham and WT RAS mice. Although the heart weight of db/db RAS mice was significantly higher than that of db/db sham mice at late time point, this difference was not significant after correction for body weight (Table 1). However, the heart weight of WT RAS mice corrected for body weight was significantly greater than that of WT sham mice corrected for body weight at early time point (Table 1). At both time points, the heart weight to body weight ratio in WT sham mice was significantly greater than that of db/db sham mice (Table 1).

**Table 1:** Mean ± SEM body weight, heart weight and the heart-to-body weight ratio of WT and db/db mice groups.

Mice group	Early time point			Late time point		
	Body weight (g)	Heart weight (mg)	Heart/body weight ratio	Body weight (g)	Heart weight (mg)	Heart/body weight ratio
WT RAS	18.9 ± 0.5^a^	134.4 ± 4.5	7.1 ± 0.2^d^^,^^e^	23.7 ± 0.5^a^	146.8 ± 3.5^c^	6.2 ± 0.2^d^
db/db RAS	32.5 ± 0.8^a^	140.0 ± 3.1	4.4 ± 0.1^d^	38.6 ± 1.4^a^^,^^b^	183.1 ± 9.1^c^	4.9 ± 0.3^d^
WT Sham	21.1 ± 0.2^a^	125.0 ± 3.7	5.9 ± 0.2^e^^,^^d^	25.8 ± 0.4^b^	143.3 ± 5.0	5.6 ± 0.1^e^
db/db Sham	33.3 ± 1.2^a^	131.4 ± 6.3	4.0 ± 0.1^e^	32.7 ± 1.6^b^	131.4 ± 5.8^c^	4.2 ± 0.3^e^

**Notes.**

a *p* < 0.001 = Body weight comparison between db/db RAS vs WT RAS at both early and late time points, WT sham vs db/db sham early time point.

b *p* < 0.05 = Body weight comparison between WT sham vs db/db sham late time point, db/db RAS vs db/db sham at late time point.

c *p* < 0.001 = Heart weight comparison between db/db RAS vs WT RAS at late time point, db/db RAS vs db/db sham at late time point.

d *p* < 0.001 = Heart-to-body weight ratio comparison between db/db RAS vs WT RAS at both early and late time points, WT sham vs db/db sham early time point.

e *p* < 0.05 = Heart-to-body weight ratio comparison between WT sham vs db/db sham late time point, WT RAS vs WT sham early time point.

### db/db mice with RAS had a higher mortality rate than WT mice with RAS

**Figure 1:**
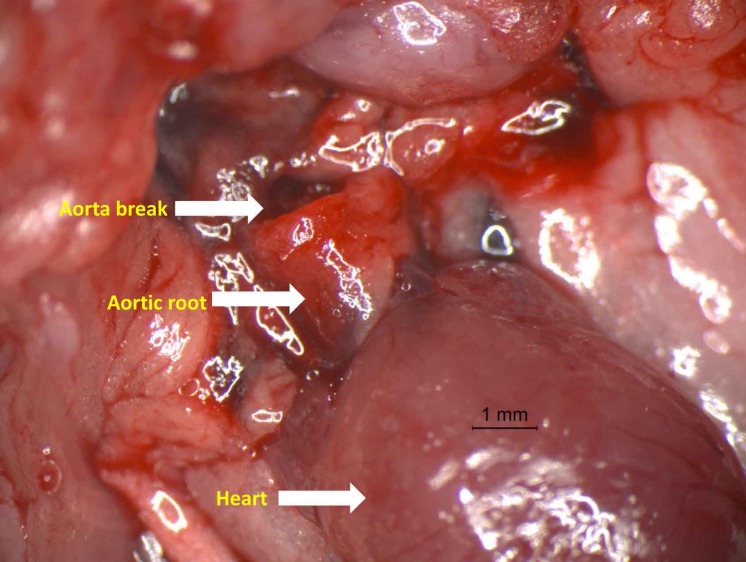
Photograph of aorta dissection showing the severe rupture just distal to aortic root. Representative photograph of aortic rupture. In the study, most aortic ruptures were found in the ascending aorta.

Fifteen of 64 (23.4%) db/db mice subjected to RAS died suddenly, whereas only 1 death was observed in 64 WT mice with RAS (1.5%, *p* < 0.001). Mortality of db/db mice as a function of time following RAS surgery is summarized in Table 2. Necropsy, performed on 7 of the db/db mice that died suddenly, showed massive intrathoracic hemorrhage with evidence of aortic dissection. The dissections appeared to occur in the ascending aorta (Fig. 1) or the proximal descending thoracic aorta. The aorta of the WT mouse that died suddenly was without grossly apparent or histopathologic abnormality. Approximately 60% of deaths in the db/db mice occurred in the two weeks following RAS surgery (Table 2).

**Table 2:** Number of mortality observed in WT and db/db mice as function of time. db/db RAS mice showed the highest mortality.

	Days following surgery												
Mice group	1	3	4	5	7	9	14	28	40	61	90	104	117
db/db/ RAS	1	2	1	2	2	0	1	1	1	1	1	1	1
db/db/ Sham	0	0	0	0	0	0	0	0	0	0	0	0	0
WT/RAS	0	0	0	0	0	1	0	0	0	0	0	0	0
WT/Sham	0	0	0	0	0	0	0	0	0	0	0	0	0

### Severity of aortic histopathologic lesions was greater in db/db RAS mice than WT RAS mice

**Figure 2:**
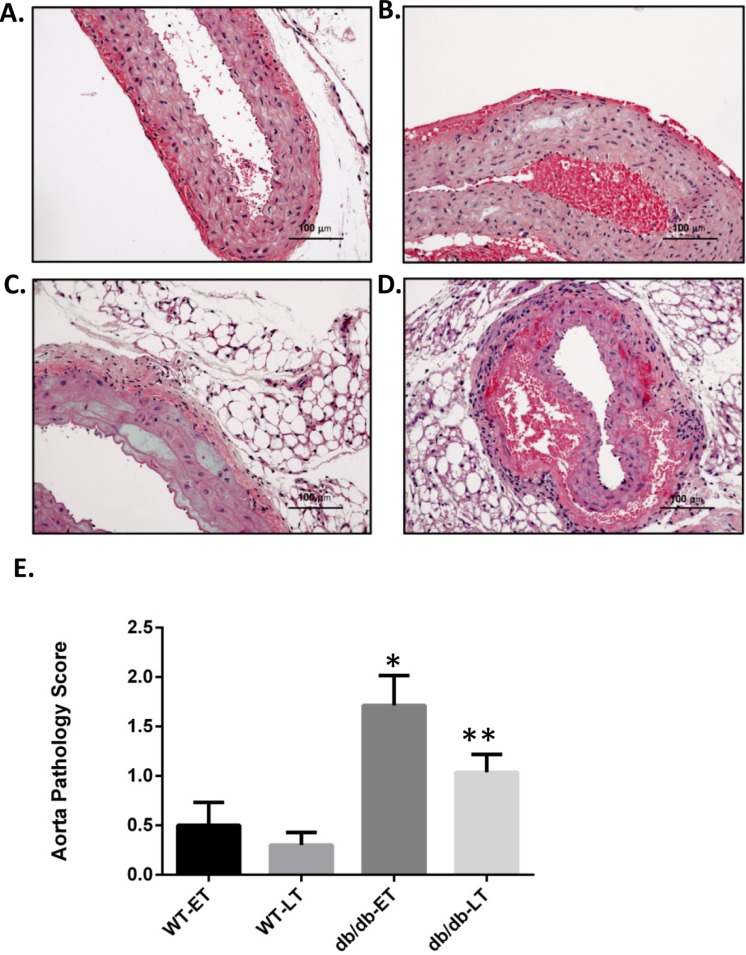
db/db mice showed higher mean pathology score at both early and late time points. Representative images of aorta illustrating semiquantitative histologic assessment scores. (A) Shows normal aorta (score of 0). (B) Focal myocyte dropout (Score of 1). (C) Multifocal myocyte dropout (Score of 2). (D) Medial disruption and hemorrhage (Score of 3). Mean pathology score of db/db and WT early and late time points animals subjected to RAS surgery. ^∗^*p* = 0.007, ^∗∗^*p* = 0.0034 in comparison to WT group.

**Figure 3:**
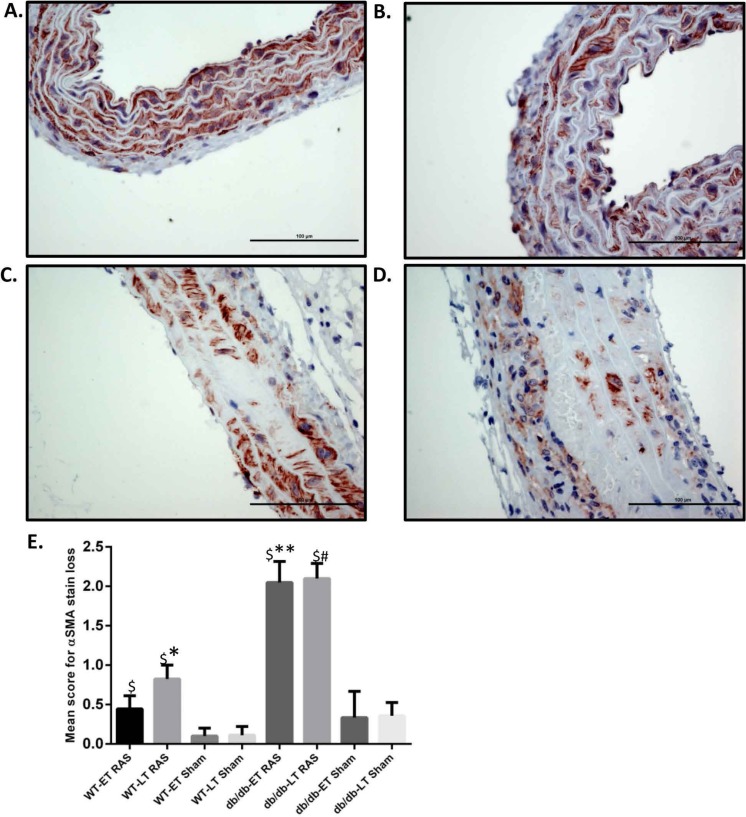
db/db mice showed more *α* smooth muscle actin loss in aorta at both early and late time points compared to WT. Representative images of aorta illustrating semiquantitative assessment of *α* smooth muscle actin (*α* SMA) loss at 400× using *α* SMA staining. (A) shows no loss of *α* SMA stain (score of 0). (B) Focal loss of *α* SMA stain (Score of 1). (C) Multifocal loss of *α* SMA stain (Score of 2). D. extensive loss of *α* SMA stain (Score of 3). (E) Mean *α* SMA stain loss score of db/db and WT early and late time points animals. ^∗^*p* = 0.0152, ^∗∗^*p* = 0.002, ^#^*p* = 0.0001 in comparison to respective sham groups. ^$^*p* = 0.0001.

Aortas were obtained for histopathologic analysis on 38 WT RAS mice (18 at week 1, 3 at week 4, 10 at week 6, and 7 at week 17), 19 WT sham mice (5 at week 1, 5 at week 2, 5 at week 6, and 4 at week 17), 48 db/db RAS mice (11 at week 1, 10 at week 2, 7 at week 4, 12 at week 6, and 8 at week 17), and 22 db/db sham mice (5 at week 1, 3 at week 2, 9 at week 6, and 5 at week 17. Histologic sections were obtained from the ascending aorta and proximal descending thoracic aorta, sites where grossly apparent aortic dissections were observed. If no aortic abnormalities were grossly apparent, a standard section of ascending aorta was obtained. A semiquantitative scoring system was employed to assess severity of aortic pathology, including medial smooth muscle dropout, medial disruption, hemorrhage, or necrosis; slides were read in a blinded fashion. Given that a majority of the sudden deaths in db/db mice with RAS occurred within the first 2 weeks following surgery, we compared histopathologic alterations in the aorta of mice studied at 2 weeks or less following surgery with those in the aorta of animals studied at greater than 2 weeks following surgery. No damage was found in the aortas of any db/db or WT sham mice. Aortic damage was observed in both db/db and WT RAS mice. Typical histopathologic alterations included medial smooth muscle dropout and medial disruption with hemorrhage (Figs. 2A–2D). At both early and late time points, db/db RAS mice showed a significantly increased aortic pathology score compared to WT RAS mice (Fig. 2E). Loss of aortic medial smooth muscle cells was associated with local or generalized reduction in medial alpha-smooth muscle actin expression (Fig. 3). Loss of alpha smooth muscle actin expression was significantly correlated with aortic pathology score in db/db mice subjected to RAS and studied at early time points (*r*^2^ = 0.62, *p* ≤ 0.0001). Alpha smooth muscle actin scores did not significantly correlate with aortic pathology scores in db/db RAS mice studied at late time points or in WT RAS mice studied at early or late time points.

### Increase in Aortic wall width observed in damaged aortae in both WT and db/db RAS mice

**Figure 4:**
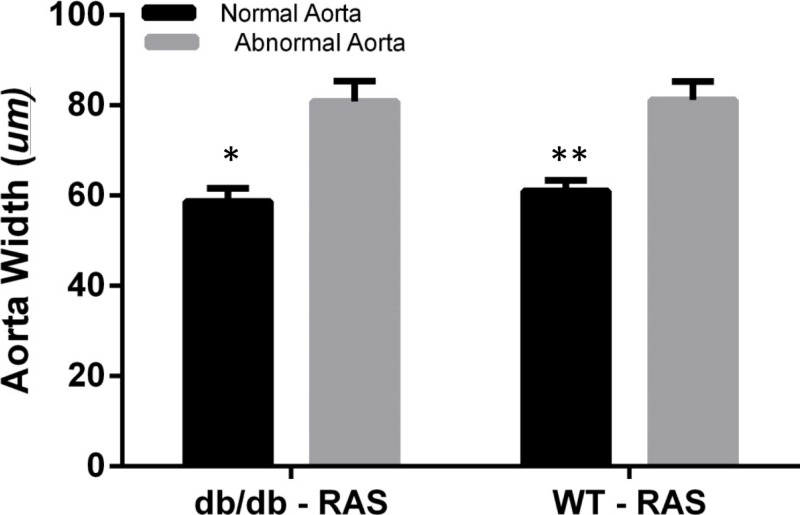
Abnormal aorta showed increased overall wall thickness. Mean aortic medial thickness was greater in aortas with histopathological scores of 1, 2, or 3 versus a score of 0 in both WT and db/db mice (^∗^*p* = 0.002, ^∗∗^*p* = 0.0001).

To determine whether aortic histopathologic alterations were associated with increased medial thickness, the aortic width was examined at 200×magnification, from the internal elastic lamina to the adventitia in WT and db/db RAS mice. Aortas demonstrating normal histopathology (score = 0) were compared with those showing isolated smooth muscle dropout (score = 1), multifocal smooth muscle dropout (score = 2) or evidence of medial disruption, hemorrhage, or necrosis (score = 3). The wall thickness of aortas showing minor to severe histopathologic abnormalities was significantly greater than that of aortas showing normal histopathology, in both db/db and WT RAS mice (*p* = 0.002 for db/db and *p* = 0.000 for WT) (Fig. 4).

### db/db RAS mice showed more cardiac fibrosis

**Figure 5:**
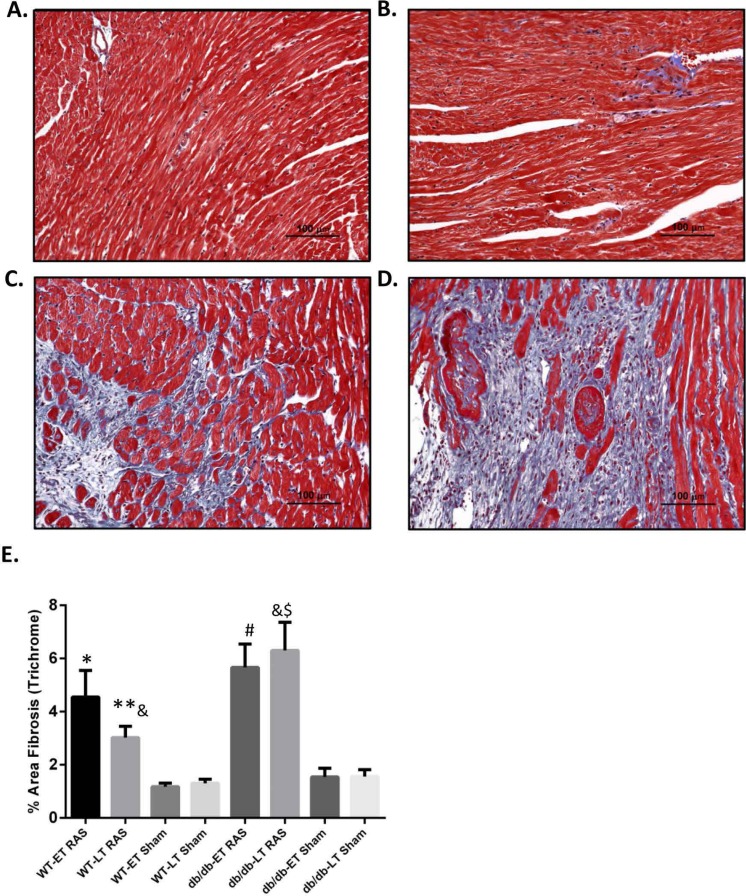
Increased cardiac fibrosis in db/db RAS mice compared to WT at later time points. Myocardial fibrosis was assessed by quantitative image analysis of trichrome stained sections at 200× magnification obtained from (A) WT sham, (B) db/db sham, (C) WT RAS and (D) db/db RAS mice (E). The mean percentage of fibrosis in db/db and WT mice at early and late time points. Both WT and db/db showed increase % fibrosis following RAS compared to their respective sham at both early and late time points. db/db RAS mice had significantly more fibrosis compared to WT RAS at late time points. ^∗^*p* = 0.02, ^∗∗^*p* = 0.009, ^#^*p* = 0.001, ^$^*p* = 0.0001 compared to their respective shams and ^&^*p* = 0.03.

Myocardial remodeling was determined as the percentage of surface area staining blue with trichrome stain, as assessed by computer-assisted quantitative morphometric analysis. Representative photomicrographs are shown in Figs. 5A–5D. Cardiac fibrosis was increased in both db/db and WT RAS mice compared to their sham at both early and late time points (Fig. 5E). There was a significantly higher degree of fibrosis in db/db RAS mice compared to WT RAS mice at time points greater than 2 weeks (Figs. 5E). The extent of cardiac fibrosis did not correlate with aortic pathology.

## Discussion

We unexpectedly found an increased prevalence of aortic dissection leading to sudden death in db/db but not WT mice subjected to RAS. Aortas showed more medial smooth muscle dropout, medial disruption and hemorrhage in db/db mice than WT mice with RAS. Aortic lesions were observed in the ascending and proximal descending aorta, which are common sites for human aortic dissection.

Although a well-established risk factor for atherosclerosis, diabetes is associated with a reduced prevalence of atherosclerotic aortic aneurysms (Takagi & Umemoto, 2015; Golledge, Cooper & Chai, 2015; Pafili et al., 2015; Pafili, Gouni-Berthold & Papanas, 2015). It is thought that production of advanced glycation end products and extracellular matrix deposition may lead to reduced macrophage infiltration, matrix metalloproteinase activity, and plasmin activation (Pafili et al., 2015). In the current study, we did not find any significant difference in aortic medial thickness between db/db and WT mice subjected to RAS. Furthermore, we did not find any evidence of atherosclerosis in the db/db mice. In order to reduce potential complications associated with delayed wound healing in older db/db mice, we performed RAS surgery on young (6–7 weeks old) mice, prior to the development of end organ manifestations of diabetes.

Aortic aneurysms and dissection result from either genetic or acquired defects in the aortic wall. Since aortic aneurysms are typically asymptomatic until they rupture, it is important to better characterize the pathophysiology of aortic aneurysms and to identify patients who are at increased risk of developing these catastrophic lesions. Several animal models of aortic aneurysms have been developed to elucidate basic mechanisms underlying the development of these lesions (Baker et al., 1984).

Ang II infusion in atherosclerotic Apolipoprotein E (Apo-E) deficient mice has been employed as a model of aortic aneurysms (Baker et al., 1984; Van Kats et al., 1998; Daugherty et al., 2010). In addition to increasing blood pressure, Ang II promotes influx of T cells and macrophages into the aorta and other vessels (Lindpaintner & Ganten, 1991). Ang II infusion promotes abdominal aortic aneurysms independent of increased blood pressure in hypercholesterolemic mice (Qian & Montgomery, 2012). These studies suggest that the pro-inflammatory effect of Ang II is more important than its hypertensive effect in the development of aortic aneurysms (Qian & Montgomery, 2012). Along these lines, the incidence of both atherosclerosis and of aortic aneurysms is significantly reduced in Apo-E deficient mice lacking CCR2, a critical receptor that directs influx of macrophages and T cells to sites of tissue injury (Daugherty et al., 2010). Although endogenous angiotensin II production is likely responsible for the development of aortic lesions in mice with RAS, the dissections observed in the current study were observed primarily in the ascending and proximal descending aorta, whereas angiotensin II infused mice tend to develop abdominal aortic aneurysms (Baker et al., 1984; Qian & Montgomery, 2012; Clauser, 1998). Mononuclear cell infiltrates were observed in both WT and db/db RAS mice, in accordance with studies indicating that angiotensin II promotes vascular inflammation (Klingbeil et al., 2003; Harrison et al., 2010). However, we did not observe any significant differences in the severity of mononuclear cell infiltrates between WT and db/db RAS mice in this study.

Recent studies have defined a critical role for TGF-*β* signaling in the pathogenesis of aortic aneurysms and dissection. TGF-*β* signaling is initiated through binding of TGF-*β* to the type 2 receptor (TBR2), recruitment of the type 1 receptor (TBR1), followed by phosphorylation of SMAD3, recruitment of SMAD4, nuclear translocation and activation of target genes (Cheng & Grande, 2002; Cheng & Grande, 2009). Mutations in SMAD3, have been identified in up to 2% of patients with familial thoracic aneurysms leading to acute aortic dissection (Regalado et al., 2011) Patients with the Loeys-Dietz syndrome have mutations in receptors for TGF-*β* (TGFBR1 and TGFBR2) (Loeys et al., 2006).

In mice with homozygous deletion of the Smad3 gene, angiotensin II (Ang II) infusion promotes the development of aortic aneurysms and aortic dissection. Development of aneurysms is due to Ang II mediated macrophage infiltration and upregulation of NOS2 (inducible nitric oxide synthase), matrix metalloproteinases (MMP) 2 and 9 rather than hypertension alone (Tan et al., 2013). Of note, we did not observe any aortic dissections in our previous study employing RAS in mice bearing homozygous deletion of the Smad3 gene (Warner et al., 2012).

In addition to the increased risk of developing ischemic heart disease, patients with diabetes are prone to develop diabetic cardiomyopathy, characterized by cardiac hypertrophy, myocardial fibrosis, and diastolic dysfunction (Asbun & Villarreal, 2006) Although leptin-deficient db/db mice do not develop myocardial remodeling or cardiac dysfunction, they are more susceptible to Ang II mediated hypertrophy and dysfunction (Harrap et al., 1996). Along these lines, we observed more severe cardiac remodeling in db/db RAS mice than WT RAS mice. Smad3 null mice crossed with leptin deficient db/db diabetic mice were protected from the development of diabetic cardiomyopathy (Biernacka et al., 2015). However, db/db SMAD3 null mice showed increased mortality due to spontaneous rupture of the ascending aorta (Biernacka et al., 2015). SMAD3 deficiency was associated with increased MMP-2 and MMP-9 activity, with no change in tissue inhibitor of matrix metalloproteinase-1 (TIMP-1) activity.

In mice fed a high fat diet or obese ob/ob mice, Ang II infusion promotes macrophage influx into the aorta and fosters the development of aortic aneurysms (Clauser, 1998). In our model, which employs endogenous activation of the renin-angiotensin system due to renal artery stenosis (Warner et al., 2012; Wang et al., 2013; Cheng et al., 2009) we find that db/db mice are more susceptible to both renal and cardiovascular disease than WT mice, despite similar elevation in systolic blood pressure (Hartono et al., 2014). In our previous study of db/db mice subjected to RAS, we found that angiotensin I production is elevated in both db/db and WT mice, but returns to baseline levels by 6 weeks following surgery (Hartono et al., 2014), making it unlikely that differences in blood pressure or angiotensin II production are responsible for the development of aortic dissection in db/db mice.

Although we believe that this is the first study to document aortic lesions in db/db mice subjected to renovascular hypertension, there are several limitations. First, this was a retrospective study which was not designed to identify aortic lesions. It was not possible to perform histopathologic analysis on many of the mice that died suddenly. Nevertheless, we were able to identify more aortic lesions in db/db mice, even in vessels with no grossly apparent pathology. Finally, we have not established a potential mechanism through which hyperglycemia/diabetes interacts with renovascular hypertension to produce the aortic lesions. This may be at least in part due to the fact that many, if not most, of the aortas obtained from both db/db or WT mice had minor histopathologic abnormalities. Although an effort was made to sample grossly abnormal regions of the aorta, the focal nature of the lesions may lead to an underestimation of the degree of histologic abnormalities, including macrophage infiltration.

Placement of a cuff on the right renal artery produces kidney lesions that recapitulate many of the histopathologic features of human renal artery stenosis (Keddis et al., 2010). The stenotic kidney of mice with RAS develops progressive tubular atrophy, interstitial inflammation, and interstitial fibrosis (Wang et al., 2013; Cheng et al., 2009), whereas the contralateral kidney undergoes compensatory enlargement with minimal histopathologic abnormalities. Unlike WT mice, db/db mice subjected to RAS develop bilateral, progressive renal disease, with severe atrophy of the stenotic kidney and diffuse mesangial sclerosis, with segmental and global glomerulosclerosis, interstitial fibrosis, and tubular atrophy—features reminiscent of diabetic nephropathy. Of note, the severity of renal or cardiac lesions did not correlate with aortic pathology. Future studies will determine whether the diabetic phenotype interacts with the pro-inflammatory state driven by elevated Ang II levels and will define maladaptive signaling pathways triggered through which hyperglycemia/diabetes intersect with renovascular hypertension to produce aortic dissection.

## Supplemental Information

10.7717/peerj.1736/supp-1 Figure S1 Data used for the Figs. 2, 3 and 4E

10.7717/peerj.1736/supp-2 Data S1 Raw dataset used for the paper

## Additional Information and Declarations

### Data Availability

The following information was supplied regarding data availability: Data can be found in the Supplemental Information.
